# An injectable copolymer of fatty acids (ARA 3000 BETA) as a promising treatment for osteoarthritis

**DOI:** 10.1038/s41598-023-34206-8

**Published:** 2023-05-13

**Authors:** Karine Toupet, Christian Jorgensen, Danièle Noël

**Affiliations:** 1 IRMB, University of Montpellier, INSERM, Montpellier, France; 2 ECELLFRANCE, University of Montpellier, INSERM, CHU Montpellier, Montpellier, France; 3 Clinical Immunology and Osteoarticular Diseases Therapeutic Unit, Hôpital Lapeyronie, Montpellier, France

**Keywords:** Rheumatic diseases, Pharmaceutics

## Abstract

Osteoarthritis (OA) is the most prevalent rheumatic disease and a fast growing cause of disability. Current pharmacological treatments include antalgics and non-steroid anti-inflammatory drugs to control pain and inflammation as well as slow acting drugs such as intra-articular (IA) administration of hyaluronic acid. Oral supplementation or diet rich in polyunsaturated free fatty acids are proposed but evidence for benefit is still under debate. We here investigated the therapeutic potential of ARA 3000 BETA, an injectable copolymer of fatty acids, at the structural level in OA. Collagenase-induced osteoarthritis model was induced in C57BL/6 mice by collagenase injection into knee joint. Mice were treated with one or two IA or four intra-muscular injections (IM) of ARA 3000 BETA. At sacrifice, knee joints were recovered for cartilage analysis by confocal laser scanning microscopy (CLSM) and bone analysis by micro-computed tomography system. OA histological scoring was performed after safranin O/fast green staining. Histological analysis revealed a protective effect against cartilage degradation in treated knee joints after IM and IA administration. This was confirmed by CLSM with a significant improvement of all articular cartilage parameters, including thickness, volume and surface degradation whatever the administration route. A slight protective effect was also noticed on subchondral bone parameters and knee joint calcification after IM administration and to a lesser extent, two IA injections. We demonstrated the therapeutic efficacy of injectable ARA 3000 BETA in OA with a protection against cartilage and bone alterations providing the proof-of-concept that clinical translation might be envisioned to delay disease progression.

## Introduction

Osteoarthritis (OA) is the most prevalent chronic joint disease that mostly affects fingers, knees and hips. The number of patients is constantly increasing with the ageing of the population and the disease management cost is increasing as well. OA is characterized by cartilage degradation, bone sclerosis, osteophyte formation, meniscal and ligament calcification and, synovial inflammation leading to joint pain and functional impairment. Development of OA is correlated with an increased production of matrix metalloproteinases (MMPs)-1, -3 and -13 and of A Disintegrin And Metalloproteinase with Thrombospondin motifs (ADAMTS)-4, -5 and of pro-inflammatory cytokines, including interleukin (IL)-1β, -6 and tumor necrosis factor (TNF)-α^1^. Current treatments use antalgics and non-steroid anti-inflammatory drugs (NSAID) but are only symptomatic and prescribed to alleviate pain and reduce inflammation. With no efficient treatment to stop the disease, a number of studies aim at investigating new therapeutic agents.

In the past decades, dietary fatty acids (FAs) have gained much interest due to their critical role in essential signaling cascades maintaining cellular functions and tissue homeostasis while imbalanced fat composition may lead to joint inflammation and induce OA (for review, see^2^). FAs can be classified into saturated (SFAs), monounsaturated fatty acids (MUFAs) and polyunsaturated fatty acids (PUFAs), which include omega-6 (n-6) and omega-3 (n-3) PUFAs. It is now accepted that SFAs and n-6 PUFAs have pro-inflammatory functions, whereas n-3 PUFAs are described as anti-inflammatory FAs. FAs have been found in serum, synovial fluid, cartilage and subchondral bone where they exert different effects on fibroblasts, osteoblasts and chondrocytes^3–5^. Indeed, n-3 PUFAs are beneficial for cartilage metabolism by reducing inflammatory factors, including prostaglandin E2 (PGE2), cyclooxygenase (COX)-2, IL1β, TNFα and decreasing ADAMTS-4, MMP3^6–9^. Beneficial effects of n-3 PUFA supplementation in diet on the progression of OA and reduction of associated pain were demonstrated in various animal models, such as the diet-induced obesity murine model^10^, the destabilization of medial meniscus murine model^11,12^, the anterior cruciate ligament transection model in rabbits^13^, dogs with hip or stifle OA^14–17^ or OA-prone Dunkin-Hartley Guinea pigs^18^. In humans, some studies have investigated the link between dietary PUFAs and clinical signs of OA. A positive relationship was observed between n-6 PUFAs and OA risk while inverse association was noticed between n-3 PUFAs and synovitis^19^. Beneficial effects of n-3 PUFAs were also reported on pain and function^20,21^.

The most abundant FAs in the synovial fluid are linoleic acid (n-6 PUFA), oleic acid (n-9 MUFA) and palmitic acid (SFA), representing 80% of total FAs^7^. Interestingly, addition of oleic acid or palmitic acid in chondrocyte cultures inhibited MMP1 expression and GAG release by cartilage explants, suggesting an anti-inflammatory function of these FAs. In the early 1980s, a copolymer of oleic acid, palmitic acid and stearic acid, called ARA 3000 BETA, has been developed as an injectable formulation by the company LEXMOOR (Saint-Rémy-de-Provence, France). The in vivo effect of ARA 3000 BETA was first evaluated in OA patient dogs and shown to decrease pain and lameness^22^. It is still widely used for OA treatment in dogs and cows. In vitro studies have demonstrated the anti-inflammatory properties of ARA 3000 BETA on human chondrocytes with a reduction of nitric oxyde (NO), PGE2 and MMP production and the inhibition of the NF-kB pathway^23^. In vivo, intra-muscular injection of ARA 3000 BETA, one day before inflammatory adjuvant-induced arthritis initiation in rats, reduced clinical signs, including edemas, ankylosis and weight loss^23^. However, no data proved the efficacy of ARA 3000 BETA over a placebo control in a relevant model of OA, even though it seems to have a great interest to control joint inflammation and reduce cartilage destruction.

Here, we evaluated the therapeutic effect of local injections of ARA 3000 BETA in the collagenase-induced osteoarthritis model (CIOA), which is described as the reference model of inflammatory osteoarthritis^24,25^. The main objective of the study was to determine the effect of a single or repeated ARA 3000 BETA dose in the intra-articular space of knee joints. Intra-muscular administration was evaluated as a positive control related to the current veterinary practice. Effects of the drug were evaluated at the structural level using quantitative histomorphometric parameters of cartilage and bone tissues by confocal laser scanning microscopy and microcomputed tomography, respectively. The study will set the basis for translation of ARA 3000 BETA into the clinics for human OA.

## Methods

### Reagent

The copolymer of FAs ARA 3000 BETA was obtained by combination of oleic acid (8.75 mg/mL), palmitic acid (5.4 mg/mL) and stearic acid (4 mg/mL). The copolymer formulation was produced and supplied by LEXMOOR Laboratory.

### Collagenase-induced osteoarthritis model

All animal procedures were approved by the Ethical Committee for animal experimentation of the Languedoc-Roussillon, France before initiation of the study (approval APAFIS#5351-2016050919079187). Animal procedures were performed in accordance with the European guidelines for the care and use of laboratory animals (2010/63/UE) and in accordance with ARRIVE guidelines. Local injections and euthanasia were performed after anesthesia with isoflurane gas, and all efforts were made to minimize suffering. Mice were housed in solid bottomed plastic cages in quiet rooms at 22° ± 1 °C, 60% controlled humidity, and 12 h/12 h light/dark cycle. Animals were used after an adaptation period of 7 days and had free access to tap water and standard pelleted chow. In vivo experiments were done on 10 week-old male C57BL/6 mice obtained from Janvier (St. Berthevin, France). Collagenase-induced osteoarthritis model (CIOA) was induced by two injections of 1 U type VII collagenase (Sigma-Aldrich) in 5 µL saline into the intra-articular space of the right knee joint of mice at day 0 and 2 using a syringe with a 20G needle, as described in^25^. Collagenase-treated mice were divided into 4 groups of 15 mice: (1) mice received 5 µL saline by intra-articular (IA) route in right knee joints at day 7 (control OA group) and contralateral left knee joints received two injections of saline at days 0 and 2 (control healthy knees); (2) mice received two IA injections of 5 µL of ARA 3000 BETA in the right knee joint at day 7 (group IA1); (3) mice received a single IA injection of 5 µL of ARA 3000 BETA in the right knee joint at days 7 and 21 (group IA2) and (4) mice received 50 µL of ARA 3000 BETA by intra-muscular (IM) route in the right *Anterior tibialis* at days 3, 11, 18 and 25 (group IM). Mice were distributed in cages: 10 mice/cage from two groups were mixed to limit cage-associated bias. The utility of referring to contralateral knees as healthy control knees was reported elsewhere^26^ and justified by the reduction of animal number as recommended by ethical committees. As OA control, we used the injection of saline because ARA 3000 BETA consists of a polymer co-formulated with its excipients, which does not allow to separate the therapeutic drug from its excipients. Fifteen animals per group was calculated to be required to demonstrate significance at the 5% level with a power of 80% using the G*power software. Mice were euthanatized at day 42 and hind paws were recovered and fixed in 4% formaldehyde at room temperature for 48 h, for further analysis.

### Bone parameter analyses

After fixation, hind paws were scanned in a micro-computed tomography system (µCT SkyScan 1176, Bruker). Each scan was performed according to the following parameters: 0.5 mm Aluminium filter, 50 kV, 500 µA, pixel size of 18 µm and 0.5° rotation angle. Images were then reconstructed using NRecon software (SkyScan NRecon version 1.7.4.6, Bruker). Misalignment compensation, ring artefacts and beam-hardening were adjusted to obtain a correct reconstruction of entire knee joint. Bone degradation was quantified in subchondral bone plate and epiphysis region on medial and lateral plateaus of each tibia using CTAn software (version 1.17.7.2, Bruker). Osteophyte formation and meniscal/ligament calcification were quantified on entire knee joints. Reconstructed 3D images of knee joints were obtained using Avizo Lite software (version 2019.3, FEI).

### Confocal laser scanning microscopy

For each hind paw, tibia and femur were separated and smooth tissues were carefully removed to denude articular cartilage. Articular cartilage of tibial medial and lateral plateaus was scanned separately through their depth in XYZ-mode, with a confocal laser scanning microscope (CLSM; TCS SP5-II, Leica Microsystems). The following parameters were used to image each sample: 5 × dry objective, UV laser light source (l¼ 405 nm), detector gain of 800 V and line average of 5. For each sample, cartilage autofluorescence was acquired every 5 µm of depth. The resulting image stack was used to reconstitute 3D-images of each plateau, which were analysed to quantitatively evaluate the volume, thickness and surface degradation of articular cartilage. Assessment of cartilage morphometric parameters was performed in both lateral and medial plateaus of each tibia using Avizo Lite software (version 2019.3, FEI).

### Histological analysis

After CLSM analysis, tibias were decalcified in 5% formic acid at room temperature for one week and processed for histology. Samples were embedded in paraffin and frontal sections were cut (3 slices of 7 µm each 100 µm; first section at 50 µm below the cartilage surface) and stained with safranin O/fast green to analyze cartilage and bone tissues. Degradations were quantified for lateral and medial tibial plateaus using the modified Pritzker OARSI score by grading and staging each degradation as described^27^. Three sections of each sample were scored and the maximal score was assigned to the sample. Minimal score is 0, indicating absence of cartilage or bone damage, whereas 30 is the maximum score indicating important cartilage and bone degradation.

### Statistical analyses

Statistical analysis, graphs and created images were performed with GraphPad Prism 9 Software. Normal distribution and variance homogeneity of values were determined with Shapiro–Wilk and Fisher test followed by unpaired t-test. Statistical analysis compared healthy and treated groups to the OA control group. Data are presented as the mean + SEM, with p < 0.05 (*), p < 0.01 (**), p < 0.001 (***), p < 0.0001 (****).

## Results

### ARA 3000 BETA protects articular cartilage from degradation

**Figure 1:**
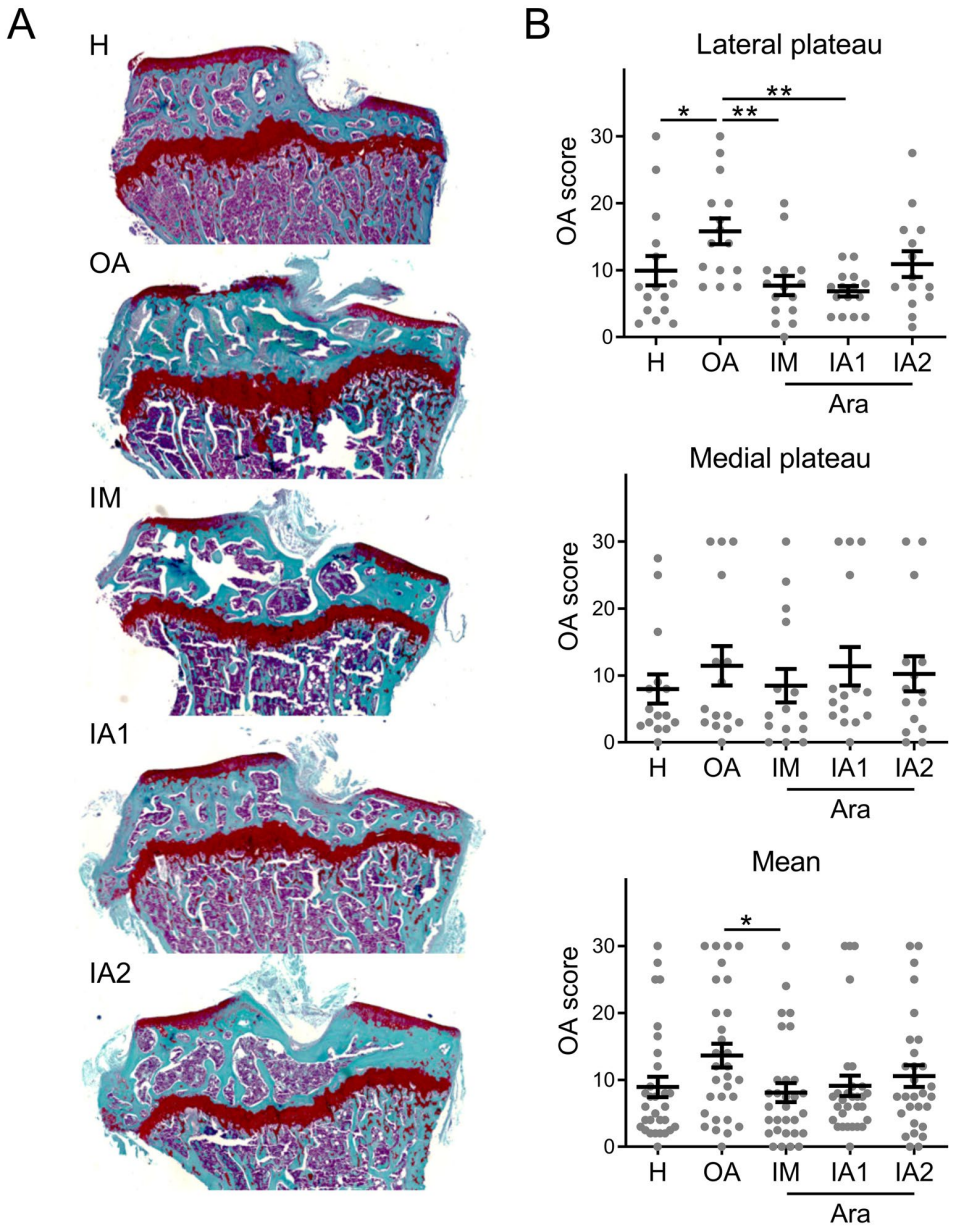
ARA 3000 BETA improved OA histological scores. (**A**) Histological images of healthy (H) mice and CIOA mice not treated (OA) or treated with four intra-muscular (IM) injections or one (IA1) or two intra-articular (IA2) injections of ARA 3000 BETA (Ara). (**B**) OA score of histological sections of tibial lateral or medial plateaus or the mean score of knee joints. Results are expressed as the mean ± SEM; *p < 0.05; **p < 0.01 (Kruskal–Wallis test with Dunn’s post-test as compared to OA control; n = 15 mice/group).

The therapeutic effect of ARA 3000 BETA was evaluated after local injection, either via the IA or IM route, in the CIOA murine model. The IM route is currently used in veterinary practice and offers the advantages of both easy access and possible repeated injections. Local IA administration is the preferred route for OA treatment allowing higher accessibility of the drug but repeated injections can be deleterious when applied on small joints such as in the mouse. We therefore compared the effect of one (IA1) or two IA (IA2) injections of ARA 3000 BETA and four repeated IM injections on OA parameters. Histological analysis is the reference method to visualize and quantify cartilage degradation by attributing an OA score, which increases with the degree of cartilage alterations (Fig. 1A). Here, the OA score significantly increased in the tibial lateral plateaus of OA knee joints compared to control healthy ones (Fig. 1B). Cartilage degradation was more obvious in the lateral tibial plateaus in regard to medial tibial plateaus, which showed no significant change. A protective effect against cartilage degradation was observed in OA knee joints treated with ARA 3000 BETA both after IM administration and IA injection, as seen with a lower OA score in the lateral plateau and total joint, with a similar tendency on the medial plateau (Fig. 1A,B). However, the most significant results were obtained after IM administration and one IA injection in the lateral plateau.

**Figure 2:**
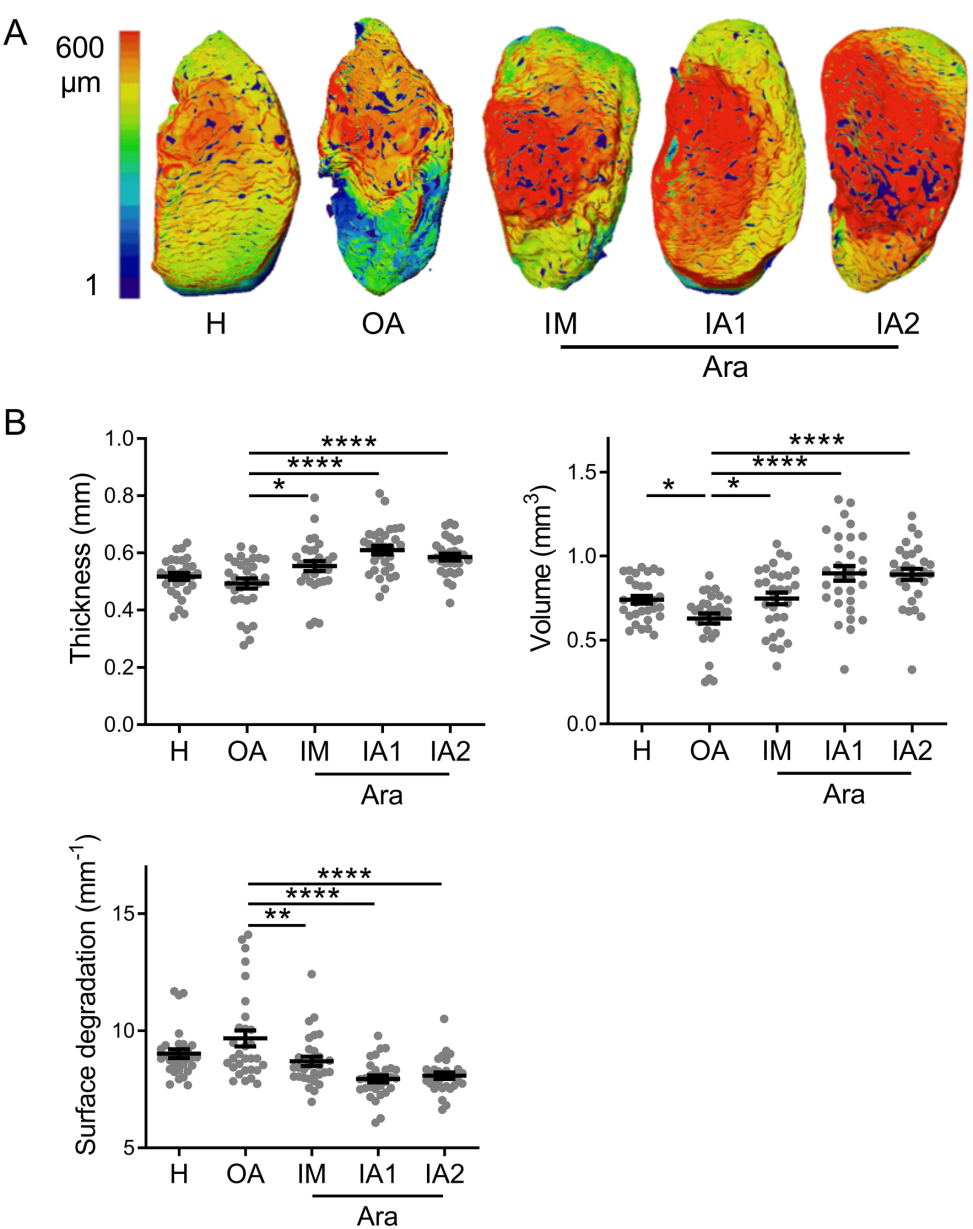
ARA 3000 BETA protected articular cartilage from degradation. (**A**) Representative 3D reconstructed images of lateral tibial cartilage of healthy (H) mice and CIOA mice not treated (OA) or treated with four intra-muscular (IM) injections or one (IA1) or two intra-articular (IA2) injections of ARA 3000 BETA (Ara) after CLSM analysis. On the left, colour code for cartilage thickness. (**B**) Histomorphometric analysis of 3D images of articular cartilage measuring thickness, volume and surface degradation. Results are expressed as the mean of tibial and median plateaus ± SEM; *p < 0.05; **p < 0.01; ****p < 0.0001 (ANOVA test with Dunett’s post-test as compared to OA control; n = 15 mice/group).

One limitation of histological analysis is the 2D visualization of a limited number of sections of knee joint that does not allow accurate quantitative analysis of articular cartilage in a whole 3D cartography. To circumvent this limitation, we performed an histomorphometric analysis of the lateral and median tibial cartilage plateaus by CLSM. Representative 3D images of lateral tibial plateaus illustrated important cartilage degradations in OA knee joints compared to healthy controls, as shown by wide areas with blue and green colours indicative of low thickness (Fig. 2A). Histomorphometric analysis of the two tibial plateaus confirmed a decrease of articular cartilage thickness and volume while surface degradation was increased in OA knee joints although not reaching significance (Fig. 2B). Interestingly, OA mice treated with ARA 3000 BETA, either by IM or IA routes, showed a significant improvement of all parameters of articular cartilage, including thickness, volume, and surface degradation compared to non-treated OA knee joints (Fig. 2A,B). No difference was observed between mice treated with one single or two IA injections of ARA 3000 BETA while a trend for higher cartilage preservation was noticed in IA injected mice versus IM administration (Fig. 2B). The chondroprotective effect of ARA 3000 BETA was therefore confirmed, whatever the route of administration.

### ARA 3000 BETA prevents knee joint calcification and subchondral bone alterations

**Figure 3:**
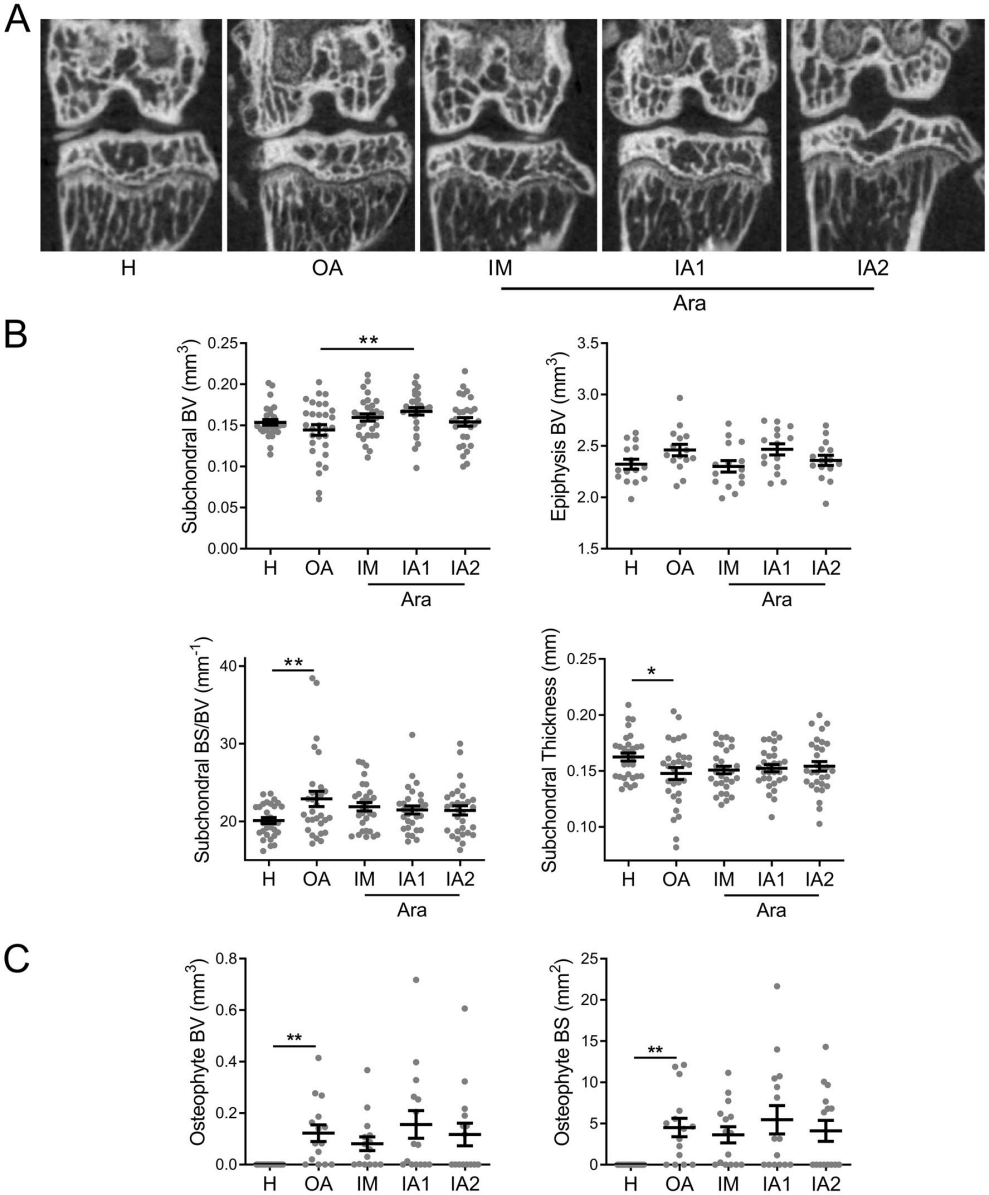
ARA 3000 BETA protected bone from OA-associated alterations. (**A**) Representative images of knee joint bone tissues in healthy (H) mice and CIOA mice not treated (OA) or treated with four intra-muscular (IM) injections or one (IA1) or two intra-articular (IA2) injections of ARA 3000 BETA (Ara) after micro-CT analysis. (**B**) Histomorphometric analysis of epiphysis bone volume (BV) indicative of sclerosis, sub-chondral bone volume (BV), thickness and bone surface/bone volume (BS/BV) parameters, indicative of bone surface alteration at the interface with cartilage. (**C**) Histomorphometric analysis of osteophyte bone volume (BV) and surface (BS) at the edges of cartilage in knee joints. Results are expressed as the mean ± SEM; *p < 0.05; **p < 0.01 (ANOVA test with Dunn’s post-test as compared to OA control; n = 15 mice).

OA is no more considered as a cartilage disease but as a disease affecting all knee joint compartments, and in particular bone. The effect of ARA 3000 BETA was therefore analysed on histomorphometric parameters of different tibial bone regions by µCT: subchondral bone plate and epiphysis. We observed sclerosis of the epiphyseal region in OA knee joints compared to healthy knee joints, which was mainly located in the medial compartment and correlated with a trends toward increased epiphyseal bone volume (Fig. 3A,B). Although not significant, OA knee joints treated with one IM or two IA doses of ARA 3000 BETA tended to be protected against epiphyseal sclerosis. At the subchondral bone level, we observed a decrease of bone volume and thickness and, a significant increase of bone surface degradation (BS/BV parameter) in OA knee joints compared to healthy knee joints (Fig. 3B). The subchondral bone volume was higher in all treated groups compared to OA knee joints but statistical significance was obtained only with a single ARA 3000 BETA IA injection. We also noticed a trend towards lower bone surface degradation in all treated groups, whatever the route and number of injections (Fig. 3B). Osteophyte formation is another hallmark of OA as measured by a highly significant increase of bone volume and surface at the edges of articular cartilage in OA knee joints (Fig. 3C). No significant reduction in osteophyte size was seen in the knee joints treated with ARA 3000 BETA, although a trend towards lower volume and surface was noticed after IM or two IA administrations.

**Figure 4:**
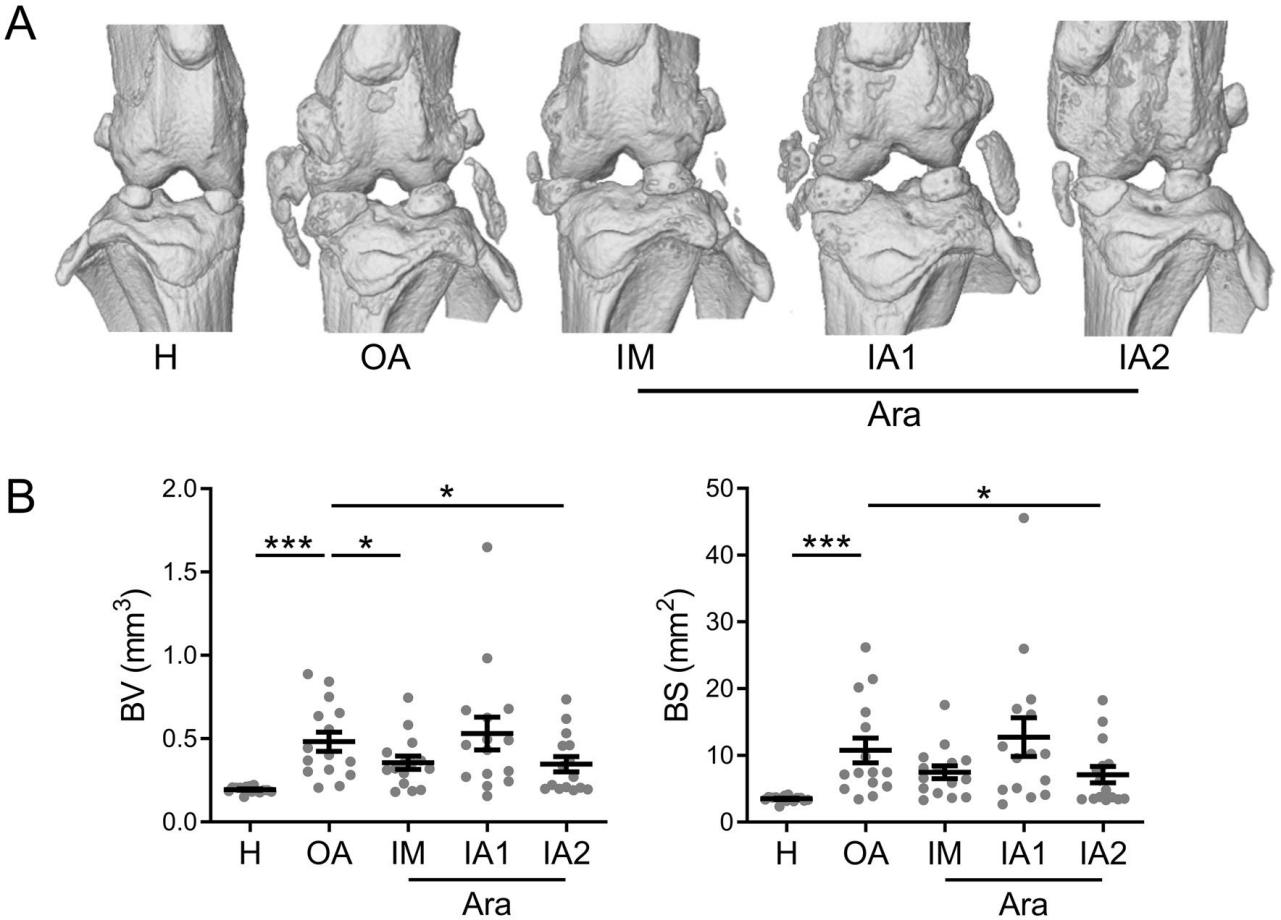
ARA 3000 BETA protected knee joint soft tissues from OA-associated calcification. (**A**) Representative 3D reconstructed images of knee joints in healthy (H) mice and CIOA mice not treated (OA) or treated with four intra-muscular (IM) injections or one (IA1) or two intra-articular (IA2) injections of ARA 3000 BETA (Ara) after micro-CT analysis showing mineralized menisci and external ligaments. (**B**) Histomorphometric analysis of bone volume (BV) and surface (BS) of mineralized tissues in knee joints. Results are expressed as the mean ± SEM; *p < 0.05; ***p < 0.001 (ANOVA test with Dunn’s post-test as compared to OA control; n = 15 mice).

Finally, abnormal calcification of menisci as well as lateral and median ligaments was observed in OA knee joints compared to healthy knee joints (Fig. 4A). This was confirmed by a significant increase of bone volume and surface (BV and BS) parameters, respectively, characterizing the calcification of these non-mineralized tissues (Fig. 4B). Of note, BV and BS parameters were significantly lower in OA knee joints treated with ARA 3000 BETA either after one IM or two IA injections. Altogether, the data indicated a protective effect of ARA 3000 BETA on subchondral bone volume after one IA injection and on knee joint calcification after IM administration and two IA injections.

## Discussion

Here, we demonstrated that ARA 3000 BETA strongly protects against articular cartilage degradations while its effect on bone alterations was slighter in the CIOA murine model, which is representative of inflammatory osteoarthritis. While the study cannot extrapolate what could be the effect of ARA 3000 BETA in other pathotypes of the disease, it might be anticipated that the anti-inflammatory effect claimed could be also observed in other pathotypes where inflammation is observed. This protective effect was reported not only with standard histological analysis on representative knee joint sections but also with accurate histomorphometric analysis of whole tissues thanks to CLSM and µCT technologies.

The present study underlined the IA route as the most efficient for cartilage preservation while both IA and IM administration tends to improve epiphyseal sclerosis and subchondral bone parameters and, reduced meniscus and peri-articular ligament calcification. Indeed, a single IA injection of 5 µL of ARA 3000 BETA at day 7 was efficient to reduce cartilage degradation for at least the 5 weeks of follow-up. By comparison, the four IM injections of 50 µL of ARA 3000 BETA resulted in less significant improvement of cartilage parameters indicating that local injections were more efficient. By CLSM analysis, we observed higher thickness and volume of articular cartilage in treated knee joints compared to healthy controls suggesting a possible regenerative effect of ARA 3000 BETA. However, this result was not validated by histological analysis, which revealed minor lesions in healthy knee joints as indicated by the mean OA score. The occurrence of lesions in the contralateral knee joints has already been described in OA murine models^28,29^. This suggests that ARA 3000 BETA protects cartilage from degradation but does not induce regeneration. The slight bone protective effect of ARA 3000 BETA was mostly observed on bone volume preservation after a single IA administration while two IA injections were more efficient to reduce intra-articular calcifications. We injected a 20-fold larger volume of compound by IM than IA, likely explaining the effectiveness of treatment. Both IM and IA routes of administration are commonly used to efficiently deliver medication in various models of osteoarthritis, osteonecrosis, osteoporosis and bone injuries^30–34^. The present study therefore underlines the interest of low doses of ARA 3000 BETA and IA administration for protecting cartilage and, to a lesser extent bone, from OA-related alterations.

This is the first study demonstrating the beneficial role of an injectable formulation of FAs in OA. Previous studies reported a therapeutic effect of ARA 3000 BETA in a placebo-controlled study on inflammatory adjuvant-induced arthritis in rats suggesting an anti-inflammatory effect^23^. Two studies have also reported encouraging results after three IM injections at one week intervals of ARA 3000 BETA in 20 and 22 patient dogs with OA^22,35^. Improvement of pain and function was noticed by the veterinarian with no improvement on structural radiological parameters. However, in absence of placebo controls, efficacy of ARA 3000 BETA could not be proven. Up to now, the therapeutic effect of FAs on OA progression was evaluated in animal models by supplementation in the diet^36^. Diet supplementation with omega-3 PUFA-rich fish oil in dogs with OA improved lameness, functional disability and reduced discomfort compared to dogs with normal diet^14,16,37,38^. By contrast, the population-based NEO study reported a positive association of SFA and PUFA plasma concentrations and clinically defined hand OA and structural knee OA in men but not in women^39^. This is the first study evaluating the association between FA levels and clinical or structural OA in animals that needs to be confirmed by further studies. By using an injectable formulation of three FAs-containing ARA 3000 BETA, we demonstrated that IM or IA administration of FAs improves histological and structural signs of OA. Furthermore, the wide use of ARA 3000 BETA in veterinary practice for dogs with OA provides evidence for beneficial effect of the drug with no reported adverse effects (https://lexmoor.com). A transient pain sensation at injection time and slight swelling at the injection site have been reported in dogs. At the macroscopical level, the present study revealed a slight swelling of the muscle, which is likely related to the volume of injection but no behavioral pain was observed after treatment. Therefore, the present study provides the proof-of-concept that translation to human could be envisioned.

Although the mechanism of action of ARA 3000 BETA is not understood, its chondroprotective effect was demonstrated on primary human chondrocytes, which released lower amounts of MMP-1, MMP-3, MMP-13, NO and PGE2^23^. Similar data were reported after incubation of oleic and palmitic FAs on chondrocytes and cartilage explants from OA patients^7^. Oleic and palmitic acids were taken up by the cells and a reduced expression of MMP1 and prostaglandin-endoperoxide synthase 2 (PTGS2) as well as lower glycosaminoglycans (GAG) release were observed under inflammatory conditions. Regarding the impact of FAs on bone, opposite effects have been described on osteoclastogenesis. SFAs such as palmitic acid enhance RANKL-induced osteoclastogenesis while n-9 MUFAs such as oleic acid prevent this process through activation of diacylglycerol acyl transferase 1 (DGAT1), an enzyme involved in triglyceride synthesis^2,40^. Depending on the relative concentration of each compound in the formulation, the overall balance between pro- and anti-osteoclastogenic effects may be likely shifted towards bone protection/regeneration. Apart from anti-inflammatory and bone preservative properties, ARA 3000 BETA formulation and its inherent viscosity might act in the intra-articular space through a viscosupplementation effect. This has not been investigated but warrants further studies in larger animals.

In conclusion, we demonstrated the therapeutic efficacy of ARA 3000 BETA, an injectable copolymer of FAs, in inflammatory OA with a significant protection against articular cartilage and bone lesions. We validated the clinical efficiency of IM and IA routes of administration but IA injections might be favoured as a single low dose efficiently protects cartilage from alterations. Although further studies are required to understand the mechanism of action of ARA 3000 BETA and its role on signalling cascades involved in OA, our results provide the proof-of-concept that clinical translation might be envisioned to stop or delay disease progression.

## Data Availability

All data generated or analysed during this study are included in this published article.
